# The Relationship between Mindful Attention Awareness, Perceived Stress and Subjective Wellbeing

**DOI:** 10.3390/ijerph182312290

**Published:** 2021-11-23

**Authors:** Stevie-Jae Hepburn, Annemaree Carroll, Louise McCuaig

**Affiliations:** 1 School of Education, The University of Queensland, Brisbane, QLD 4067, Australia; a.carroll@uq.edu.au; 2 Faculty of Humanities and Social Sciences, The University of Queensland, Brisbane, QLD 4067, Australia; l.mccuaig@uq.edu.au

**Keywords:** mindfulness, pre-service, teacher wellbeing, perceived stress, teacher stress, health

## Abstract

It has been suggested that mindfulness is a predictive factor in self-reported perceived stress. The present study aimed to investigate the link between mindful attention awareness, perceived stress and subjective wellbeing without the presence of a complementary intervention to promote mindfulness-based strategies. Methods: The online survey participants (*N* = 257) were university students enrolled in initial teacher training. Self-report measures included the Mindful Attention Awareness Scale (MAAS), Perceived Stress Scale (PSS) and Personal Wellbeing Index (PWI). Results: PWI was negatively correlated with PSS (*r* = −0.550, *p* = .001), MAAS was negatively correlated with PSS (*r* = −0.567, *p* = .001) and positively correlated with PWI (*r* = 0.336, *p* = .001). The mean score for PSS (M = 20.61, SD = 6.62) was above the reported norm (14.2). Conclusions: The findings suggest that higher levels of mindful attention awareness may be associated with lower levels of perceived stress and higher subjective wellbeing levels and lower levels of perceived stress may be associated with higher subjective wellbeing. The findings confirm that pre-service teachers are a demographic that experiences elevated levels of perceived stress regardless of the stage in initial teacher training programs.

## 1. Introduction

University students are consistently reported as a demographic that experiences elevated stress levels compared to the general population [1]. It has been suggested that universities need to provide support for students because mental health concerns can have a broad range of impacts on a student’s quality of life [2,3]. In Australia, it has been reported that pre-service teachers (PSTs) are experiencing elevated stress levels when age-matched with the general population [4,5,6]. The impact of the COVID-19 pandemic has presented additional challenges for PSTs [7,8].

Previous research has highlighted the link between natural levels of mindfulness (dispositional mindfulness) and perceived stress for university students (pre-service students) without the presence of a mindfulness-based intervention or program (e.g., [9]). Such research suggests that mindfulness as a cognitive state is a significantly strong factor in predicting stress levels. In addition, mindfulness was strongly correlated with personality aspects (extraversion, concentration and agreeableness) that assist with managing stressful events and experiences. University students reporting high levels of mindfulness reported lower levels of depression, fatigue, tension and confusion. University students with experience practising mindfulness meditation and self-compassion have higher self-reported psychological wellbeing and lower perceived stress and depression [10]. The present study aimed to contribute to the existing research by addressing the following research question: Is there a relationship between perceived stress, natural levels of mindfulness (attention awareness) and subjective wellbeing?

### 1.1. Background

#### 1.1.1. Stress Levels for Pre-Service Teachers

Concerns have been raised regarding the level of reported stress experienced by university students. In a survey of university students (*N* = 13,984) by the World Health Organization [11] 35% of respondents screened positive for at least one lifetime mental health disorder and 31% screened positive for at least one 12-month mental health disorder (measured by the Diagnostic and Statistical Manual of Mental Disorders—DSM-IV). The range of estimates for 12-month and lifetime mental health disorders was 48.3–43.3% in Australia to 22.4–19.1% in Belgium. Major depressive disorder was the most common across all countries, followed by generalised anxiety disorder. The stress levels experienced by university students were demonstrated in an Australian study (*N* = 6479) where 67.4% of students were aged between 18 and 24 years [6]. When age-matched with the general population (Australian data figures), 19.2% of the students reported very high levels of distress, whereas only 3% of the general population were in the high or very high distress range. The elevated psychological distress levels reported by the students (83.9%) were significantly higher than the general population (29%, Australian data figures reported in [6]).

Similar findings were reported by Geng and colleagues [4] when examining the stress levels of Early Childhood, Primary and Secondary PSTs (*N* = 291). The general population sample reported stress levels between 14.52–17.23, all of the PSTs enrolled in Initial Teacher Training (ITT) in the study, reported higher stress levels than the general population. Higher levels of stress were reported on the first placement and students experiencing high levels of stress received lower overall performance ratings, while students that established a positive working relationship with their supervising teachers reported lower overall levels of stress. When investigating the experiences of postgraduate (*n* = 151) and undergraduate (*n* = 159) PSTs, Geng and colleagues [5] reported postgraduate PSTs perceived stress (measured with the Perceived Stress Scale; [12] were significantly higher than undergraduate PSTs and that the two groups experienced different stressors. In a recent study including students from the Faculties of Health and Education in an Australian university (*N* = 552), the education students (*n* = 172) shared stressors included study commitments, academic stress, work-life balance, practicum structure, financial pressure and interactions with supervising staff on practicum [13]. During practicum placement, PSTs are assessed by a supervising teacher and required to provide a portfolio demonstrating evidence of their ability regarding the national professional standards for teaching from the Australian Institute of Teaching and School Leadership (AITSL [14]). In addition, PSTs are required to complete a Teaching Performance Appraisal (e.g., Graduate Teaching Performance Assessment).

Regarding PST training programs, concerns have been raised surrounding inconsistencies between program structure, practicum experience and the level of support provided by supervising teachers and mentors [15]. Wilson and Huynh [16] reported in a mixed-methods study (*N* = 177) PSTs’ ability to cope is linked to the quality of the mentoring relationship. Changes in self-efficacy and stress experienced by PST have been linked to or influenced by the relationship formed with mentor teachers [17]. Professional identity can be influenced by the nature of the feedback they receive, emotional experiences on practicum, the quality of the relationships they form, and the background and experience of the PST [18]. Perceived social support was positively associated with higher psychological wellbeing levels in undergraduate psychology students (*N* = 228) [10]. A fundamental aspect of psychological wellbeing is forming positive social relationships [19,20]. Also, our ability to engage with social relationships is influenced by how we feel (subjective wellbeing) [21].

#### 1.1.2. Mindfulness and Wellbeing

As a meditative practise, mindfulness has been a prominent research topic in mainstream psychology since the 1970s [22]. Mindfulness is the process of maintaining focus on present reality [23]; it is a way of paying attention [24]. Mindfulness is most commonly referred to as “paying attention in a particular way: on purpose, in the present moment, and nonjudgmentally” [25] p. 4. It has been suggested that mindfulness allows for adaptive coping and managing stressful situations or experiences [10]. Mindfulness is also associated with improved psychological wellbeing [26]. The cultivation of mindfulness is a cognitive process that increases insight, also referred to as *insight meditation* (Buddhist: *Vipassana*) [27] p. 164. There are two main models for cultivating mindfulness, first, the 2500-year-old model based on Buddhist psychology and the teachings of Shakyamuni Buddha; second, the 30-year-old contemporary model popularised by Kabat-Zinn [28]. Both models focus on reducing suffering, improving wellbeing and increasing positive emotions [29]. The conceptualisation of Mindfulness from the Buddhist perspective emphasises the connection between attention and memory [30]. Mindfulness is a part of the Noble Eightfold Path. Mindfulness is the translation of *sati* (Sanskrit: *smrti*) that means “memory” and is closely related to *sarati* (verb) that refers to the process “to remember” [29,31]. The concern surrounding the use of the term “mindfulness” (noun) implies that it is a fixed trait, whereas the traditional Buddhist texts (*Satipatthana Sutta*) describe *sati* as a process [31]. For additional information surrounding the traditional practices of Buddhism, see Santina [32]. Unfortunately, a detailed account of the interesting history of Mindfulness is beyond the scope of the present study.

Due to the exponential growth of research surrounding mindfulness, there has been an increase in self-report measures to assess mindfulness as a stable trait (a facet of an individual’s personality). The concern raised by Grossman [31] p. 221 is that the definition of mindfulness varies widely between the scales, and there is “little or no account of the developmental and contextual aspects inherent in the Buddhist formulation” of mindfulness. There is additional inconsistency across the scales due to the varying components or facets of mindfulness [33]. Likewise, there is the issue that the scales are based on the individual’s perception of their own behaviour. Nonetheless, one of the most commonly used self-report measures is the Five Facets of Mindfulness Questionnaire (FFMQ; [34]) with five sub-scales that measure mindfulness as a stable trait. The Mindfulness in Teaching Scale [35] operates under a two-factor model with intrapersonal and interpersonal dimensions. The Kentucky Inventory of Mindfulness Skills [36] is skills-based (neither trait nor state) and determines the use of mindfulness as a skill. The most frequently used scale that produces a single score for mindfulness is the Mindful Attention Awareness Scale (MAAS; [37]). As noted above, the FFMQ explores mindfulness as a trait, whereas the MAAS relates to mindfulness as a state and focuses on present moment awareness, referred to as dispositional mindfulness. “Dispositional and state mindfulness predict self-regulated behavior and positive emotional states” [37] p. 822. The MAAS has been included in the present study because it produces a single score that assesses an individual’s tendency to enter the state of mindfulness, that is, their natural level of mindfulness. The aim was not to measure the facets of mindfulness; therefore, the FFMQ was not included. The FFMQ has been included in numerous studies examining the use of mindfulness-based interventions for stress management (see, [38,39,40,41,42,43,44]).

Wellbeing is a concept that is multifaceted, dynamic and at times, contested [45]. Subjective wellbeing refers to satisfaction with life and the presence of positive effect, also referred to as the *hedonic* approach [46]. In comparison, the *eudemonic* approach includes life domains, for example, the Psychological Theory of Wellbeing [19]. A commonality across wellbeing constructs is that they have a subjective experiential nature [47] and wellbeing is defined by a collection of indicators, not a single indicator [48]. See the review by Cooke and colleagues for a review of the measures of wellbeing [49]. There is bidirectional feedback between psychological and physiological wellbeing not to mention influential factors such as, neurochemistry, brain activity, social support, genetics, and personality traits [50]. For example, decreased amygdala activation when evaluating negative information has been associated with higher subjective well-being levels [51].

#### 1.1.3. The Stress Response and Mindfulness-Based Techniques

The stress response is an intricate adaptive response to ensure an organism’s survival in a threatening situation. Threats (real or perceived) are detected within the brain by the amygdala, the hippocampus and the prefrontal cortex. The hippocampus activates memories from previous experiences and can override the amygdala, whereas the prefrontal cortex uses meta-cognition and attention (volition) to override the amygdala [52]. Stress is known to manifest in two ways; first, it can alter an individual’s behaviour and decisions surrounding their health (for example, diet and lifestyle choices) and second, stress can trigger alterations in physiological systems thus increasing the risk of disease [53]. Physiological stressors are considered stressors that “start out in the body” and psychological stressors “start out as ideas, fears, and sources of anxiety and that only later become part of the workings of the body” [54] p. 67. The physiological changes undertaken to ensure an organism’s survival involve powerful neuroendocrinal changes within the body and equally powerful emotional changes [54] p. 72. The emotions most commonly associated with the stress response are anxiety as anticipation or apprehension in the absence of a threat, anger as an outwardly focused, reactive impulse and fear generated in the presence of the threat [54]. Chronic stress and trauma can trigger anxiety symptoms and play a role in the development of anxiety-related conditions such as general anxiety disorder, panic disorder, and acute stress disorders, which impact sleep patterns, functioning and productivity at work, relationships, and create feelings of fear, panic and pessimistic thoughts [55]. The ability to appraise or assess a potential stressor and re-appraise stressors relates to the transactional stress model [56]. Individuals react to stressors in the environment and consider their ability to cope. The ability to re-appraise and respond rather than react has been linked to mindfulness-based strategies (see [57]). It has been suggested that ITT courses or degrees provide little opportunity for PSTs to recognise potential coping strategies and the signs of stress [58].

It has been suggested that mindfulness training can support and foster stress resilience pathways in the brain (see [59]). Positive emotions have been associated with mindfulness-based meditation (e.g., loving-kindness practice), and the reported findings have included increased mindful attention awareness (mindfulness as a state) (see [60]). In addition, positive emotions have been associated with endocrine changes that down-regulate the stress response (HPA-axis activation). For example, oxytocin is released in positive social interactions, decreasing the stress response [61]. Mindfulness-based meditation has been linked to increased salivary oxytocin levels in psychology university students (*N* = 68) [62]. As evidenced by Fredrickson (see [63,64,65,66]), positive emotions can provide an antidote to the physiological changes that occur as a result of the arousal caused by negative emotions (e.g., stressors). Positive emotions support the development of an individual’s social, cognitive, physical and psychological resources [61,64]. Mindfulness-based approaches are increasing as complementary therapies for mental health concerns, for example, mindfulness-based cognitive behavioural therapy [67]. Similarly, mindfulness and attending to the present moment are recommended to improve physical wellbeing, such as a complementary therapy for diabetes. It is suggested that mindfulness increases awareness of lifestyle factors and behaviours [68]. It has also been suggested that mindfulness training can increase interoception and proprioception (awareness of the body) measured by heartbeat perception accuracy (HBPa) which may support physical wellbeing (see [69]).

There is extensive research examining the impact of mindfulness-based interventions (MBIs) in clinical and non-clinical settings and previous research has examined the link between mindfulness as a state and perceived stress (e.g., [9]). However, there is limited research examining the influence of mindful attention awareness on subjective wellbeing. The present study intended to contribute to the existing research and identify the relationship between mindful attention awareness (cognitive state), perceived stress, *and* subjective wellbeing for PSTs without the presence of an intervention or program.

## 2. Materials and Methods

### 2.1. Participants

The study formed part of a larger doctoral research project investigating PSTs’ perceived stress, wellbeing, and mindful attention awareness (see [70]). For inclusion in the study, the participants needed to be enrolled in either an undergraduate or postgraduate initial teacher training (ITT) degree (e.g., Bachelor of Primary Education or Master of Teaching) or a dual degree (e.g., Bachelor of Arts and Education). Participants that were not enrolled in ITT degrees were excluded from the study. Of the 392 participants that consented to complete the survey, 257 valid responses were recorded. The participant characteristics are included in Table 1. Incomplete responses were excluded. Of the 241 participants that indicated their age, 82.2% were between 20–29 years, 5.4% between 30–34 years and the remaining 12.4% were between 35–59 years.

### 2.2. Procedure

The study protocol followed the National Statement on Ethical Conduct in Human Research and was approved by The University of Queensland Human Research Ethics Committee. The survey instruments were formatted in Qualtrics [71] and a participant information letter and informed consent was included in the survey design. The survey took approximately 15-min to complete and included 80 questions in total. The survey link was distributed to full-time students enrolled in ITT degrees. The survey list was emailed to students at two universities by the student services department. At a third university, it was displayed in a voluntary research newsletter and at the final two universities, it was distributed to students by the course coordinators. The research team did not have contact with the student cohorts included in the study. The participants were required to open the email and click on the survey link, they were directed to the survey management system to complete the survey.

Due to the differences in the degree structure and practicum requirements across universities in Queensland, the participants were asked to indicate the year of study they had completed. The responses were grouped as “first-year”, “mid-degree” and “final-year”. The easily identifiable groups were the “first year” and “final year” and due to the varying degree lengths, participants in either their second or third year were coded as “mid-degree”. The participants in their second and third year of study were grouped as “mid-degree”, and the mid-year category (*n* = 116) formed the largest sample, followed by participants in their final year of study (*n* = 91) and the first-year participants (*n* = 50).

### 2.3. Measures

Mindful Attention Awareness Scale (MAAS). Attention Awareness was measured by the MAAS [37], which has been included in previous studies to examine the application of contemplative and emotion training for registered teachers [72] and integrating mindfulness training into teacher education programs [73,74]. In addition, the MAAS has been used in mindfulness-based interventions for psychology students [62] and working adults [60]. Brown and Ryan developed the 15-item self-report measure to assess the link between mindfulness and wellbeing. The MAAS does not produce sub-scales (facets) unlike other mindfulness scales. The MAAS is scored to produce an overall score for attention awareness (mindfulness). The MAAS has a 6-point Likert scale and uses indirect items; for example, *I find myself preoccupied with the future or the past.* The use of indirect items can be more diagnostic for examining the naturally occurring characteristic of mindfulness. The internal consistency levels range from .80 to .90 with high test-retest reliability, known-group validity, and convergent validity [37].

Personal Wellbeing Index (PWI). The International Wellbeing Group developed the PWI from the Comprehensive Quality of Life Scale (ComQOL) [75]. To date, no known teacher wellbeing studies have included the PWI. Previous studies have examined the psychometric equivalence of the adult and child PWI (e.g., [76]) and reported the PWI measures the same construct for subjective wellbeing in both populations. The seven ComQOL domains have been validated as an accurate measure of life satisfaction and subjective wellbeing [77]. The eight items in the PWI represent the seven quality of life domains: *health*, *relationships*, *safety*, *community*-*connectedness*, *future security*, *achievement in life* and *standard of living*. The optional eighth domain of *spirituality/religion* was included. The 11-point End-Defined Response Scale does not rely on adjectival descriptors (i.e., 0 = no satisfaction at all to 10 = completely satisfied). In Australia and overseas the Cronbach alpha lies between .70 and .85. An overall subjective wellbeing score can be derived from the sum of the scale items, or each domain can be scored separately [78]. In the present study, an overall subjective wellbeing score was calculated.

Perceived Stress Scale (PSS). The PSS [12] is an established self-report measure used to assess if individuals feel their lives are stressful, overwhelming, and unpredictable. The 5-point Likert scale (0 = never, to 4 = fairly often) includes ten items to measure psychological distress (e.g., *In the last month, how often have you felt that you were unable to control the important things in your life?*). The higher the overall (summed) score, the higher the perceived stress level. The PSS coefficient alphas ranged from .77 to .78 [44]. “Average stress” levels are reported as a score of 13 and “high stress” as >20. The PSS has been included as an evaluative measure in MBIs for teacher wellbeing [79,80], PST wellbeing [73,74,81] and PST stress [4,5,82]. The PSS has been included in MBIs in the health sciences [83,84,85].

### 2.4. Data Analysis

The survey responses were exported to the IBM Statistical Package for the Social Sciences (SPSS) Version 27 (IBM, Armonk, NY, USA). The scales were re-coded and scored as per the author’s instructions. Descriptive and inferential statistics were completed for all datasets, and the data met the required assumptions for completing a linear regression analysis to address the research questions (i.e., univariate normality, linear relationship, homoscedasticity). To identify if there was a relationship between mindful attention awareness (independent variable) and perceived stress and wellbeing (dependent variables) a multiple regression was not completed and a separate regression analysis was completed for each dependent variable [86]. A regression analysis was completed to determine if perceived stress (independent variable) influenced wellbeing.

## 3. Results

There was a high level of internal consistency (Cronbach’s alpha above .70) for the Mindful Attention Awareness Scale (α = .88); Perceived Stress Scale (α = .88); Personal Wellbeing Index (α = .86).

The normative mean range for the MAAS is reported as 4.2 (SD = 0.69) in community adults (*N* = 436) and 3.83 (SD = 0.70) in college-aged students (*N* = 2277) [37]. In the present study, the participant sample mean (M = 3.56) falls below the adult and community norm range reported by the MAAS authors (summary in Table 2). The normative mean range for the PWI for Western countries is 70–80 points and for Australia 73.4–76.4 points [78]. The participant sample mean (M = 56.78) is below the reported norm average.

### 3.1. Percevied Stress Levels for Pre-Service Teachers

Of the 246 participants, 50% reported a PSS score above 21. Approximately 25% of the participants reported a score lower than 16, and approximately 25% of the participants reported a score higher than 25. The PSS scores were determined for each sub-sample: ‘first year’, ‘mid-degree’ and ‘final year’ (see Table 3).

Sixty per cent of the first-year group, 68% of the mid-year group and final year group were aged 20–24 years. The reported norm population score for this age group is 14.2 [87]. The norm groups from a US population sample (*N* = 2387) for the PSS are reported as 13.7 (*n =* 1406) for females and 12.1 (*n =* 926) for males [87]. The average PSS scores across all three groups were numerically above the norm population score.

### 3.2. The Influence of Mindful Attention Awareness on Perceived Stress and Subjective Wellbeing

**Figure 1:**
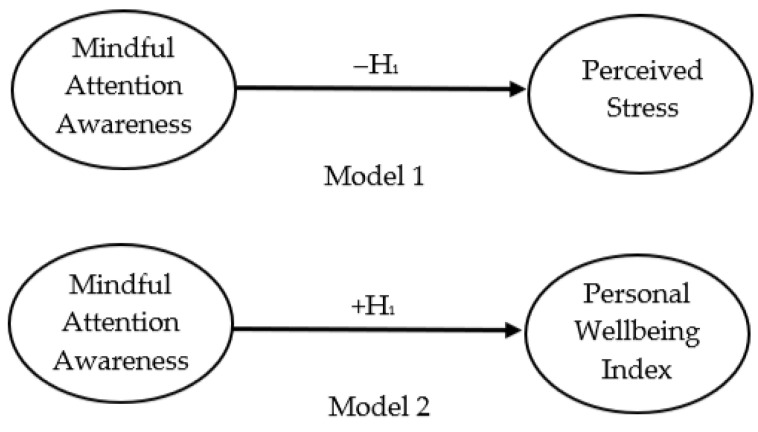
The proposed relationship between attention awareness, perceived stress and subjective wellbeing.

The regression analysis aimed to examine if a change in mindful attention awareness (MAAS, independent variable) resulted in a change (increase or decrease) in perceived stress (PSS) and wellbeing (PWI, dependent variables). Based on the literature reviewed, it was proposed that as MAAS scores increased, PSS scores would decrease due to the mechanisms of mindfulness and the impact on the arousal response (discussed above). The relationship between attention awareness, perceived stress and wellbeing is shown in model one and two (Figure 1).

With reference to the research question for the present study:

- Null hypothesis (H_0_): There is no significant relationship between attention awareness, perceived stress and subjective wellbeing.
- Alternative hypothesis (H_1_): Higher attention awareness scores will be negatively associated with perceived stress scores and positively associated with subjective wellbeing scores.

H_1_ indicates that as attention awareness scores increase, subjective wellbeing scores will increase (+H_1_), and perceived stress scores will decrease (−H_1_).

A linear regress was calculated to predict perceived stress and wellbeing (dependent variables) based on mindful attention awareness (independent variable). A significant relationship was found (*F* (1, 233) = 110.44, *p* = .001) between MAAS scores and PSS scores, with an R^2^ of 0.32 (*n* = 235). The r-squared value suggests that MAAS could explain 32% of the variation of PSS scores. The equation for the least square’s regression line is:

*Perceived Stress = 36.7 + (−4.5) × Mindful Attention Awareness*

A significant relationship was found (*F* (1, 236) = 30, *p* = .001) between MAAS and PWI, with an R^2^ of 0.11 (*n* = 238), therefore suggesting that mindful attention awareness may explain 11% of the variation of subjective wellbeing. The equation for the least square’s regression line is:

*Personal Wellbeing Index = 40.6 + 4.4 × Mindful Attention Awareness*

Based on the results from the linear regression analysis, the *p*-values were below 0.05.

### 3.3. The influence of Perceived Stress on Subjective Wellbeing

A regression analysis was completed to identify the relationship between perceived stress (PSS, independent variable) and subjective wellbeing (PWI, dependent variable).

- H_0_: There is no significant relationship between perceived stress and subjective wellbeing.
- H_1_: Higher subjective wellbeing scores will be negatively associated with perceived stress scores.

**Figure 2:**
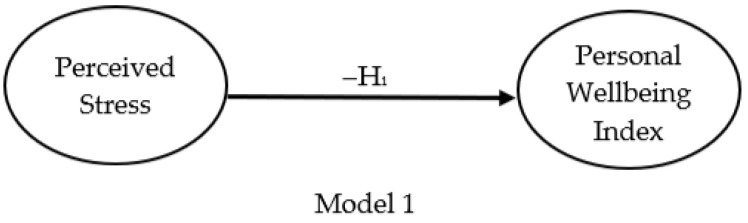
Relationship between perceived stress and subjective wellbeing.

The relationship between perceived stress (independent variable) and wellbeing (dependent variable) is shown in model three, illustrated by Figure 2. The proposed model is based on the literature reviewed that suggests perceived stress and the ability to cope with everyday stressors negatively impact subjective wellbeing. H_1_ indicates that as perceived stress scores increase, subjective wellbeing scores decrease (−H_1_).

A linear regression was completed to predict wellbeing (dependent variables) based on perceived stress (independent variable). A significant relationship was found (*F* (1, 242) = 104.8, *p* = .001) with an R^2^ 0.30 (*n* = 244). This suggests that 30% of the variation of PWI scores could be explained by PSS scores. The slope for the regression line for perceived stress and subjective wellbeing is −0.91. The negative value indicates that as PSS scores increased, PWI scores decreased. The equation for the least-squares regression line is:

*Personal Wellbeing Index = 75.58 + (−0.91) × Perceived Stress Scale*

As detailed in Table 4, the findings from this study suggest MAAS was negatively correlated with PSS (*r* = −0.567, *p* = .001), indicating a moderate negative linear association [88] between mindful attention awareness and perceived stress. This suggests that the PSS scores decreased as the MAAS scores increased (model one). Mindful attention awareness was positively correlated with subjective wellbeing (*r* = 0.336, *p* = .001), indicating a weak linear association [88] between mindful attention awareness and subjective wellbeing therefore, as MAAS scores increased, PWI increased (model two). The results suggest rejecting the null hypothesis. The findings from this study suggest PWI was negatively correlated with PSS (*r* = −0.550, *p* = .001), indicating a moderate negative linear association between perceived stress and subjective wellbeing (model 3). This result suggests that as PSS scores decreased, PWI scores increased.

## 4. Discussion

The results provided a snapshot of the education student population from five large Queensland universities. The participant sample reflected the demographics of those entering the profession (based on the Queensland College of Teachers report [89]. The participant sample was dominated by undergraduate students, reflecting the participant samples from previous studies investigating PST stress (e.g., [5]). The experience of PSTs is influenced by a myriad of factors operating at the macro (institutional) and micro (classroom) levels. An individual’s response to stress can depend on unique physiological and psychological factors and environmental conditions (e.g., placement experience, academic and family commitments) [90]. However, what is well documented is that university students experience elevated levels of psychological distress compared to the general population [4,6]. The results support the findings from similar studies with Australian university students (e.g., [4,5,6]). The results highlight that all three sub-groups were above the reported norm population score however, those nearing the end of their ITT reported higher perceived stress. This finding may relate to the fact that practicum placements feature predominantly in the final year of ITT degrees. This finding may support the previous studies that identify the practicum placement as a period of elevated stress for PST (e.g., [1,13,91,92]).

A detailed exploration of the three sub-groups was beyond the scope of the present study. This study formed part of a larger Doctoral research project and further discussion of the stress levels of PSTs over the duration of the year are reported elsewhere [70,93]. Exploring the experiences of students at different stages in their degree would provide additional information that could potentially inform teacher training programs through gaining further insight into the needs of the different cohorts—consequently providing the opportunity to assist in the development of support mechanisms for education students at every stage of their teacher training journey.

The results suggested a significant relationship between mindful attention awareness and perceived stress and a moderate relationship between mindful attention awareness and subjective wellbeing. The negative correlation between the MAAS scores and PSS scores supports Kostanski’s [4] argument that an individual reporting a high MAAS score is more likely to report a lower PSS score. Adopting a mindful perspective facilitates the ability to re-appraise situations and decrease habitual reactions [57]. Reappraisal has been identified as a mechanism that links mindfulness to health-enhancing behaviours [94]. In addition, higher levels of mindful attention awareness high are associated with the downregulation of the stress response and hormonal changes (e.g., oxytocin) [51,59].

In the present study, MAAS was positively correlated with subjective wellbeing, suggesting that higher MAAS scores are associated with higher PWI scores. However, psychological wellbeing is influenced by physiological, psychological and interpersonal factors [50]. Given the breadth of factors influencing wellbeing, it was not surprising that mindful attention awareness would account for a smaller percentage of wellbeing variation. Research suggests that higher subjective wellbeing levels can shield against perceived stress [95]. The results indicated that 30% of the variation of PWI scores could be explained by PSS scores, suggesting a significant relationship between perceived stress and subjective wellbeing. As evidenced in the results, individuals who lower perceived stress levels, reported higher subjective wellbeing. The participant sample mean for subjective wellbeing (M = 56.78) was below the reported norm average, which could relate to the high levels of perceived stress. The results suggest that there is a relationship between high natural levels of mindfulness and lower perceived stress conversely, lower MAAS scores are associated with higher PSS scores and in the present study the participant’s PSS scores were above the “high stress” level which is reflected by the lower than average (norm) MAAS scores.

PST wellbeing research indicates that teacher education training programs fail to provide adequate training on the identification and impact of stress and stress management techniques [90,96,97]. PSTs need to be provided with training surrounding strategies to manage the emotional labour of teaching to assist with buffering against the symptoms of burnout [90]. The present study’s findings suggested that individuals with high natural levels of mindful attention awareness experienced lower perceived stress and individuals experiencing lower perceived stress reported higher levels of subjective wellbeing. Based on the findings, perceived stress may have a greater influence on subjective wellbeing than mindful attention awareness. It could be suggested that mindful attention awareness may influence perceived stress, and perceived stress may influence wellbeing. Considering mindfulness is defined as a “cognitive-social-emotional ability that can be learned and developed” [98] p. 192 one could argue that introducing mindfulness-based strategies may assist with reducing perceived stress and, consequently, improve subjective wellbeing. Higher levels of mindful attention awareness (due to the presence of a mindfulness-based intervention) have been associated with increased prosocial emotions [60] and improved wellbeing [37].

### Study Limitations

Data collection methods via online surveys present limitations regarding response bias that is, a scale is based on an individual’s perception of their own behaviour [99]. There are limitations surrounding the survey distribution method, for instance, unknown refusal rates (deleted emails) [100]. Therefore, potential information could have been lost, for example, if students experiencing a high degree of stress would consider completing a student wellbeing survey a valuable use of time. The number of participants that opened the survey link and incomplete responses were recorded. Including the survey link in a newsletter listing research projects distributed to the whole student population could have reduced the participant response rate in comparison to a direct email to the individual students enrolled in education coursework subjects.

Similarly, there were two methods of distribution provided by the course coordinators, either the survey link was emailed directly to their students, or a notification of the survey was displayed via the online learning platform. Distributing a survey to pre-service teachers as part of a coursework subject would provide a larger participant sample and is a consideration for future research. Nonetheless, using an online platform to conduct a survey can provide efficient, timely access to the target audience [101].

## 5. Conclusions

PSTs are identified by the OECD [102] as a vulnerable demographic that requires our attention. The findings in the present study indicate that PSTs are experiencing elevated levels of perceived stress and decreased subjective wellbeing. Mindful attention awareness may relate to lower self-reported perceived stress and decreasing the stress response may positively influence subjective wellbeing. In light of the fact that mindfulness strategies can be taught and successfully included in teacher training curriculum, one could argue that providing PSTs with mindfulness-based strategies could positively contribute to coping strategies, decrease perceived stress and increase subjective wellbeing.

## Figures and Tables

**Table 1:** Characteristics of Study Participants (Time One *N* = 257).

Characteristic	Time One	
	%	*N*
Female	82.9	213
Male	17.1	44
First-year of study	19	50
Mid-degree (second or third year)	45	116
Final year of study	35	91
Fulltime study	90.3	232
Part-time study	9.7	24
Single Undergraduate degree	61.1	157
Dual Undergraduate degree	24.1	62
Postgraduate degree	14.8	38

**Table 2:** Summary of Mean Scores for the PSS, MAAS and the PWI.

Self-Report Measures	*N*	Mean	SD	Norm Range
MAAS	240	3.56	0.84	4.2
PWI	255	56.78	11.04	73.4–76.4
PSS	246	20.61	6.62	14.2

**Table 3:** Summary of the PSS scores for sub-sample.

Sub-Sample	*N*	Mean	SD
First year	47	18.34	5.99
Mid-degree	110	20.12	6.6
Final year	89	22.42	6.56

**Table 4:** Correlation Analysis for PSS, PWI and MAAS scores.

Self-Report Measures	Perceived Stress Scale	Personal Wellbeing Index	Mindful Attention Awareness
Perceived Stress Scale	-		
Personal Wellbeing Index	−0.550 **	-	
Mindful Attention Awareness	−0.567 **	0.336 **	-

** Correlation is significant at the 0.01 level (2-tailed).

## Data Availability

Data available on request due to restrictions. The data are not publicly available due to the conditions specified in the ethics application.
